# Inattention and hyperactive/impulsive component scores do not differentiate between autism spectrum disorder and attention-deficit/hyperactivity disorder in a clinical sample

**DOI:** 10.1186/s13229-020-00338-1

**Published:** 2020-04-25

**Authors:** Aneta D. Krakowski, Katherine Tombeau Cost, Evdokia Anagnostou, Meng-Chuan Lai, Jennifer Crosbie, Russell Schachar, Stelios Georgiades, Eric Duku, Peter Szatmari

**Affiliations:** 1 Department of Psychiatry, University of Toronto, 250 College Street, Toronto, Ontario M5T 1R8 Canada; 2 Department of Psychiatry, The Hospital for Sick Children, 686 Bay Street, Toronto, ON M5G 0A4 Canada; 3 Bloorview Research Institute, Holland Bloorview Kids Rehabilitation Hospital, Toronto, Ontario M4G 1R8 Canada; 4 Department of Pediatrics, University of Toronto, Toronto, Ontario M5G 1X8 Canada; 5 Margaret and Wallace McCain Centre for Child, Youth & Family Mental Health, Azrieli Adult Neurodevelopmental Centre, and Campbell Family Mental Health Research Institute, Centre for Addiction and Mental Health, Toronto, Ontario M6J 1H4 Canada; 6 Department of Psychiatry, The Hospital for Sick Children, Toronto, Ontario M5G 1X8 Canada; 7 Autism Research Centre, Department of Psychiatry, University of Cambridge, Cambridge, CB2 8AH UK; 8 Department of Psychiatry, National Taiwan University Hospital and College of Medicine, Taipei, 10002 Taiwan; 9 Department of Psychiatry & Behavioural Neurosciences, McMaster University, L8S 4 K1, Hamilton, ON Canada; 10 Offord Centre for Child Studies, McMaster Children’s Hospital and McMaster University, Hamilton, ON L8P 0A1 Canada

**Keywords:** Co-morbidity, ASD, ADHD, Symptoms, Gender, Principle component analysis

## Abstract

**Background:**

Although there is high co-occurrence between ASD and ADHD, the nature of this co-occurrence remains unclear. Our study aimed to examine the underlying relationship between ASD and ADHD symptoms in a combined sample of children with a primary clinical diagnosis of ASD or ADHD.

**Methods:**

Participants included children and youth (aged 3-20 years) with a clinical diagnosis of ASD (*n* = 303) or ADHD (*n* = 319) for a total of 622 participants. Parents of these children completed the social communication questionnaire (SCQ), a measure of autism symptoms, and the strengths and weaknesses of ADHD and normal behavior (SWAN) questionnaire, a measure of ADHD symptoms. A principal component analysis (PCA) was performed on combined SCQ and SWAN items, followed by a profile analysis comparing normalized component scores between diagnostic groups and gender.

**Results:**

PCA revealed a four-component solution (inattention, hyperactivity/impulsivity, social-communication, and restricted, repetitive, behaviors, and interests (RRBI)), with no overlap between SCQ and SWAN items in the components. Children with ASD had higher component scores in social-communication and RRBI than children with ADHD, while there was no difference in inattentive and hyperactive/impulsive scores between diagnostic groups. Males had higher scores than females in social-communication, RRBI, and hyperactivity/impulsivity components in each diagnostic group.

**Limitations:**

We did not formally assess children with ASD for ADHD using our research-criteria for ADHD, and vice versa. High rates of co-occurring ADHD in ASD, for example, may have inflated component scores in inattention and hyperactivity/impulsivity. A disadvantage with using single informant-based reports (i.e., parent-rated questionnaires) is that ASD and ADHD symptoms may be difficult to distinguish by parents, and may be interpreted differently between parents and clinicians.

**Conclusions:**

ASD and ADHD items loaded on separate components in our sample, suggesting that the measurement structure cannot explain the covariation between the two disorders in clinical samples. High levels of inattention and hyperactivity/impulsivity were seen in both ASD and ADHD in our clinical sample. This supports the need for a dimensional framework that examines neurodevelopmental domains across traditional diagnostic boundaries. Females also had lower component scores across social-communication, RRBI, and hyperactivity/impulsivity than males, suggesting that there may be gender-specific phenotypes related to the two conditions.

## Background

Autism spectrum disorder (ASD) and attention-deficit/hyperactivity disorder (ADHD) are both relatively common neurodevelopmental conditions [1, 2]. Despite there being no overlap in criteria between the two disorders as described in the DSM-5 [3], empirical studies have demonstrated a high level of co-occurrence between ASD and ADHD in both clinical and population samples [4]. In population-based samples, 22% (95% confidence interval, 95% CI 17-26%) of people with ASD are clinically diagnosed with ADHD, with even higher rates in clinical samples (34%, 95% CI 29-39%) [5]. In children and adolescents with ADHD, 21% (95% CI 18-24%) reach the diagnostic threshold for ASD [6]. A better understanding of the relationship between ASD and ADHD is required in order to understand the mechanism of the co-occurrence, minimize diagnostic error, and personalize treatment opportunities.

One possible explanation for the co-occurrence between ASD and ADHD is that they share common etiologies [7]. Evidence for shared genetic liability is supported by family and twin studies [8, 9], alongside general population studies [10, 11]. Twin studies have also examined the correlations between specific ASD and ADHD domains, and found a high genetic correlation between restricted repetitive behaviors and interests (RRBI) and domains of inattention and hyperactivity/impulsivity [9, 12, 13], and between social-communication and hyperactivity/impulsivity [10].

Despite evidence of possible shared etiologies between ASD and ADHD, our understanding of the underlying relationship of combined ASD and ADHD behavior symptoms is limited. Delineating the relationship between core domains of different neurodevelopmental conditions can help us better understand the nature of their co-occurrence. If the core domains underlying ASD and ADHD overlap, this could suggest either that (1) the two disorders share a common latent phenotypic construct, or (2) there is substantial measurement error or reporting bias.

A common latent phenotypic construct is found when item responses on a questionnaire are so highly correlated that they represent a common underlying domain. For example, if certain ASD and ADHD questionnaire items are highly correlated, they may load together in a principal component analysis and reveal a common underlying ASD/ADHD domain. A common ASD/ADHD domain might mean that certain ASD and ADHD traits are actually shared between the two disorders. Reporting bias can be observed when the same informant reports on both disorders, i.e., high scores on one disorder may bias an informant to rate an individual high (or low) on another disorder. A common latent phenotypic construct and measurement error may suggest an artificial co-occurrence between the two disorders. In contrast, if ASD and ADHD are associated with independent latent phenotypic constructs, this could suggest a “true” comorbidity in which measurement structure has no influence on the covariation between the two disorders.

Factor analysis techniques are data reduction techniques that decompose symptoms into their underlying constructs or dimensions. One commonly used factor analysis technique is principal component analysis (PCA). The underlying dimensions that are extracted in PCA are referred to as components [14]. It is well established from factor analysis studies that ADHD is composed of separate inattentive and hyperactivity/impulsivity domains [15, 16]. Likewise, recent factor analysis studies of ASD reveal separate social/communicative and RRBI domains [17, 18].

To our knowledge, only four studies to date have examined the latent constructs of combined ASD and ADHD symptoms in the same sample. One study used a clinical sample of children with ADHD [19], one used a clinical sample of children with ASD [20], and two used samples of children from the general population [12, 21]. Three studies found that ASD and ADHD symptoms mapped onto separate factors/components [12, 20, 21] and only one study [19] supported an overlapping three-factor solution composed of a social factor, an inattentiveness factor, and a third factor in which rigidity symptoms and hyperactive-impulsive symptoms grouped together. Given the paucity of studies on the topic and the heterogeneity in samples and methods, it is still unclear whether ASD and ADHD domains load together or onto separate domains. None of the prior studies was done on a combined sample of ASD and ADHD participants, which would provide a more robust test of the possibility that a shared latent construct underlies the co-occurrence of ASD and ADHD. No study that we are aware of has also looked into the variation in factor/component scores *across* the two diagnostic groups. Furthermore, gender-specific phenotypes of ASD and ADHD have been proposed [22, 23], and yet only one study that we are aware of has looked at variation in factor/component scores *across* gender [19].

The aims of our study were (i) to determine the principal components of combined ASD and ADHD symptoms in a clinical sample of children with a primary diagnosis of ASD or ADHD and (ii) to investigate whether there are differences in component scores across diagnosis and gender.

## Methods

### Participants

Children and youth (aged 3-20 years) with a clinical diagnosis of ASD or ADHD were recruited via the Province of Ontario Neurodevelopmental Disorders (POND) Network, Canada. Participants were included if they had a primary clinical diagnosis of ADHD or ASD, and participants and their caregivers had sufficient English comprehension to complete required testing. Participants in the current study were enrolled between 2012-2017. Ethics approval was received from each participating institution’s ethics review board.

Children with a primary diagnosis of ASD that were enrolled in the study had previously received a clinical diagnosis of autism, Asperger’s disorder/syndrome, or PDD-NOS by either a psychiatrist, clinical psychologist, developmental pediatrician, pediatrician, family physician or pediatric neurologist. Likewise, children with a primary clinical diagnosis of ADHD had previously received the diagnosis by either a psychiatrist, clinical psychologist, developmental pediatrician, pediatrician, family physician, or pediatric neurologist. Upon study enrollment, previous clinical diagnoses of ASD were confirmed using the Autism Diagnostic Observation Schedule, 2nd edition (ADOS-2) [24, 25] and Autism Diagnostic Interview-Revised (ADI-R) [26]. Previous clinical diagnoses of ADHD were confirmed using the parent interview for child symptoms (PICS) for ADHD [27]. The PICS is a semi-structured interview developed particularly for the diagnosis of disruptive behavior disorders, including ADHD. All diagnoses were made based on criteria in the DSM-IV-TR [28] or DSM-5 [3] depending on the time of diagnosis.

A total of 439 ASD participants and 425 ADHD participants had both the ASD symptom (SCQ) and the ADHD symptom (SWAN) measures completed by their caregivers. Those with any missing data on either the SWAN or SCQ were excluded (ASD: *n* = 101; ADHD: *n* = 106). We also excluded non-verbal children (*n* = 35) as these children by necessity had questions 2-7 incomplete on the SCQ. This resulted in a complete dataset of *n* = 303 in the ASD group and *n* = 319 in the ADHD group.

The number of children who were listed as having a co-occurring prior clinical ASD or ADHD diagnosis by their caregivers was also identified. Fifty-five children with a primary diagnosis of ASD were listed as having a co-occurring clinical ADHD diagnosis and 4 children with a primary diagnosis of ADHD were listed as having a co-occurring clinical ASD diagnosis. In cases of such co-occurring diagnoses, the diagnosis the participant had received first is referred to as the “primary diagnosis.” In the PCA analysis and subsequent profile analysis children remained grouped solely by their *primary* diagnosis.

Gender information was obtained from caregivers upon study enrollment. The two options provided on the patient enrollment form under “gender” were “male” and “female.” This information was used for subsequent profile analysis.

### Measures

ASD symptoms were assessed using the social communication questionnaire (SCQ) [29], a 40-item questionnaire that asks parents or caregivers to indicate the presence or absence of certain behaviors to help screen for autism. A score of 1 indicates the presence of an atypical behavior and a score of 0 indicates the atypical behavior is absent. The scale has good reliability and validity [30]. A score of 15 and above suggests the need for a formal assessment for ASD [30]. We used this score cutoff to define “caseness” in our descriptive data. Since item 1 is a language screening question it is not included in calculating the total of autistic symptoms.

ADHD symptoms were assessed using the strengths and weaknesses of ADHD symptoms and normal behavior rating scale (SWAN) [31], an 18-item questionnaire based on DSM-IV ADHD criteria. The SWAN has good reliability and validity [32, 33]. Caregivers, or other informants, rate each item on a seven-point scale with average behavior in the middle and positive and negative extremes of the behavior scored on either end. If a participant has six or more items on the two most negative extremes of behavior (“far below” or “below”) for the inattentive or the hyperactive/impulsive domain, they are determined to score within the ADHD clinical range, which we refer to as ADHD “caseness.” SWAN items were re-coded on a seven-point Likert scale from 0 to 6. For consistency in directionality with the SCQ, all items were reverse coded so that negative extremes of behavior received the highest score.

IQ was assessed with age-appropriate Weschler or Stanford-Binet scales. When more than one IQ score was available for a participant, the most recent IQ assessment was used. Adaptive functioning was assessed with the adaptive behavior assessment system (ABAS) first or second edition [34, 35]. The ABAS manual documents good validity and reliability [34]. Composite scores were determined for the conceptual, social and practical domains.

## Analysis

### Agreement between questionnaire caseness and clinical diagnosis

The level of agreement beyond chance between ASD or ADHD caseness and having a clinical diagnosis of ASD or ADHD as noted by the parent or caregiver was calculated for children with a primary diagnosis of ADHD and ASD, respectively, using Cohen’s kappa.

### Preliminary analysis

To remove redundant items, kappa was calculated between pairs of SCQ items (SCQ2-SCQ40) and between pairs of SWAN items (SWAN1-SWAN18). A kappa above 0.80 is considered “strong agreement” [36] and this value was used to remove redundant items. Kappa scores between SCQ items 24 and 25 indicated “strong agreement” (> 0.80), so item 25 was removed from the analysis. No items on the SWAN had such high kappa scores.

The correlation between SCQ and SWAN variables was examined using a correlation matrix. Tetrachoric and Pearson correlations were used to examine correlations between dichotomous (SCQ) and continuous (SWAN) variables, respectively. Biserial correlations were used to examine correlations between SCQ and SWAN items.

The following sets of items had very high correlations (> 0.80): SCQ 3 and 7; SCQ 28 and 30; SCQ 35 and 39; and SWAN 10 and 11. In all cases, the second question in the pair was excluded from further analysis to reduce multicollinearity (SCQ7, SCQ30, SCQ39, and SWAN11). SCQ9 and SCQ23 were also removed from the combined SCQ-SWAN analysis as they had a low Kaiser-Meyer-Olkin (KMO) (< 0.85) [37]. The KMO helps determine whether there is a strong correlation between variables, and therefore helps determine whether they are suitable for PCA. Low KMO values were not found for any SWAN items.

### Principal components analysis

We chose to use a PCA instead of an exploratory factor analysis as it is a simpler data reduction technique to use as a first step in understanding underlying ASD and ADHD dimensions. All principal component analyses were performed using R v3.5.1. Component scores were generated in R.

We performed a PCA on SCQ items combined with SWAN items in the combined clinical sample of children with ASD and ADHD. The KMO = 0.92 was “superb” [37], indicating sampling size and data adequacy. All KMO values for individual items were > 0.86. Barlett’s test of sphericity was significant, *χ* (1225) = 25,880, *p* < 0.01, indicating that the data were suitable to be used in a PCA.

For all PCAs, an oblique rotation (oblimin) was used to permit components to correlate with one another. To select the most appropriate principal components solution for each analysis, we considered the following criteria: (1) eigenvalues, (2) scree plots, (3) percentage of variance explained, (4) minimum number of item cross-loadings, and (5) clinical interpretability of the components.

After a component solution was derived, correlations between components were examined. We defined correlation coefficients of 0.10-0.29 as small, 0.30-0.49 as medium and ≥ 0.50 as large [38].

### Comparison of ASD and ADHD population with sample constructs

Before we could use the component scores of our combined ASD-ADHD sample PCA in a profile analysis, we needed to be confident that the components of our ASD sample and ADHD sample were similar. A PCA of the SCQ and SWAN items was performed separately in the ASD and ADHD samples. The PCAs of the ASD and ADHD samples were then compared on the number of components, the composition of the components, variance explained, and correlations between the components.

When ASD and ADHD PCAs were performed separately they revealed similar components (Supplemental Tables 3 and 4). There was no overlap between SCQ and SWAN items in the ASD group except for the loading of SCQ16, SCQ2, and SCQ17 in the “hyperactivity/impulsivity” component. There was no overlap between SCQ and SWAN items in the ADHD group except for the loading of SCQ4 in the “hyperactivity/impulsivity” component.

### Component scores

Component scores for each participant in the combined ASD-ADHD analysis were generated using *tenBerge method* in the *psych* library to preserve the oblique solution. Component scores were normalized to vary between 0 and 1 across groups using observed min/max to permit comparisons between groups and components in the profile analysis.

### Profile analysis with component scores

Profile analysis was conducted in SPSS version 22 [39] using the normalized (0-1) component scores generated in the PCA. Profile analysis aims to test whether groups have different profiles on certain measures and is considered to be a special application of the multivariate analysis of variance (MANOVA) [40]. We explored the profiles for the symptom components between clinical diagnoses (ASD, ADHD) and between genders (male, female). We considered whether the overall levels were equal, i.e., whether across the groups (diagnosis, gender) one diagnosis or gender had a higher total score than another diagnosis or gender when scores on all four symptom components were combined. We also considered whether the lines were parallel, i.e., whether across the groups (diagnosis, gender) the different diagnoses and genders were similar in profile of the four symptom components but possibly different in the level of each symptom component. We then assessed the flatness of the four symptom components, i.e., whether there was similarity in the amount of each of the four symptom components within the different groups (diagnosis, gender). The four-component scores were the within-participant contrast and both diagnosis (ASD, ADHD) and gender (male, female) were between-participants contrasts. Where profile analysis identified significant differences, individual group-wise (diagnosis, gender, and the interaction of diagnosis and gender) ANOVAs with Bonferroni correction for multiple testing (*α* = 0.05/4 = 0.0125) were used to determine specifically which groups were different for each symptom component.

## Results

### Missing vs. complete datasets

ASD participants from the complete dataset did not differ from those with missing data in terms of age, gender, SCQ score, SWAN inattention score, and SWAN hyperactivity/impulsivity score (Supplemental Table 1). There was a significant difference on IQ and ABAS scores with those with complete data having significantly higher IQ (*p* = 0.02) and ABAS composite scores for all domains (*p* < 0.01).

ADHD participants from the complete dataset did not differ from those with missing data in terms of gender, SCQ score, SWAN inattention score, SWAN hyperactivity/impulsivity score, IQ, and ABAS scores (Supplemental Table 2). ADHD children with complete data were significantly older than ADHD children with missing data (*p* = 0.03).

### ASD and ADHD samples

Descriptive data of the complete study sample is shown in Table 1.

**Table 1:** Descriptive data of study sample

	ASD (*n* = 303)	ADHD (*n* = 319)	*p value*	*Cohen’s d*
	Mean (SD)	Mean (SD)		
Age (years)	11.22 (3.43)	10.08 (2.74)	< 0.001**	0.37
IQ^a^	87.49 (24.63)	97.50 (15.91)	< 0.001**	0.48
ABAS composite scores^b^				
Conceptual	71.07 (15.36)	81.38 (14.04)	< 0.001 **	0.70
Social	70.96 (12.43)	85.68 (16.52)	< 0.001 **	1.00
Practical	64.23 (18.35)	79.49 (17.19)	< 0.001 **	0.86
SCQ score	19.96 (7.86)	7.76 (5.94)	< 0.001 **	1.75
SWAN INA score	4.72 (2.94)	5.40 (2.88)	< 0.01 **	0.23
SWAN IMP/HYP score	3.68 (2.96)	3.49 (3.10)	0.43	0.06
	*n* (%)	*n* (%)	*p value*	
Males	242 (79.87%)	253 (79.31%)	0.92	
Clinical co-occurring ASD^c^	-	4 (1.36%)	-	
Clinical co-occurring ADHD^d^	55 (18.97%)	-	-	
ASD caseness	221 (72.94%)	43 (13.48%)	< 0.001**	
ADHD caseness	161 (53.14%)	200 (62.70%)	0.02*	

*ADHD* attention-deficit/hyperactivity disorder, *ASD* autism spectrum disorder, *ABAS* adaptive behavior assessment system, *SCQ* social communication questionnaire, *SWAN* strengths and weaknesses of ADHD symptoms and normal behavior questionnaire, INA inattention, HYP/IMP hyperactivity/impulsivity

^a^30 ASD participants were missing IQ information and 193 ADHD participants were missing IQ information

^b^24 ASD participants were missing ABAS information and 131 ADHD participants were missing ABAS information

^c^13 ASD participants were missing co-occurring mental health condition information

^d^25 ADHD participants were missing co-occurring mental health condition information

*p < 0.05

**p < 0.01

### Agreement between questionnaire caseness and clinical diagnosis

While 53.14% of children with a primary diagnosis of ASD met the threshold for ADHD caseness on the SWAN, only 18.97% were listed as having a known secondary ADHD diagnosis. While 13.48% of children with a primary diagnosis of ADHD met the threshold for ASD caseness on the SCQ, only 1.36% were listed as having a known secondary ASD diagnosis. The level of agreement between ADHD caseness and having a co-occurring ADHD diagnosis by caregiver report was “slight” [36] (*κ* = 0.12) for children with a primary diagnosis of ASD. The level of agreement between ASD caseness and having a co-occurring ASD diagnosis was also “slight” [36] (*κ* = 0.07) for children with a primary diagnosis of ADHD. Most cases of ADHD or ASD identified by questionnaires were not reflected in co-occurring clinical diagnoses.

### Combined ASD-ADHD sample principal component analysis

In the combined sample of clinically diagnosed ASD and ADHD participants, a PCA was conducted on the 34 SCQ and 17 SWAN items with oblique rotation on the 622 children with a primary clinical diagnosis of ASD or ADHD. The scree plot showed an inflection occurring after 4 points, which explained 55% of the variance. All 4 components had an eigenvalue greater than 1. A four-component solution was chosen based on the scree plot, eigenvalues, proportion of explained variance, and clinical interpretability of the components.

Item loadings are shown in Table 2. The first component consisted mainly of social and communication items from the SCQ and was labeled the “social-communication” component. The second component consisted mainly of RRBI items from the SCQ and was labeled the “RRBI” component. The third component consisted of inattentive items from the SWAN and was labeled the “inattentive” component. Finally, the fourth component consisted of hyperactivity/impulsivity items from the SWAN and was labeled the “hyperactivity/impulsivity” component. There was no overlap between SCQ and SWAN items in any of the components.

**Table 2:** Pattern matrix of PCA loadings of SCQ and SWAN items in combined sample

Items	DSM-IV sub-domains	Component 1-SOC/COM	Component 2-RRBI	Component 3-INA	Component 4-HYP/IMP
SCQ 28—show things to engage interest (4/5)	SOC	0.91			
SCQ 20—talk to be friendly (4/5)	COM	0.80			
SCQ 27—reciprocates smiles (4/5)	SOC	0.80			
SCQ 29—shares things (4/5)	SOC	0.79			
SCQ 24—nod head (4/5)	COM	0.78			
SCQ 35—pretend play (4/5)	COM	0.77			
SCQ 22—points to show things (4/5)	COM	0.77			
SCQ 31—comforts others (4/5)	SOC	0.77			
SCQ 32—use gestures with sounds or words (4/5)	SOC	0.77			
SCQ 2—has to and fro conversation	COM	0.76			
SCQ 33—normal range of facial expressions (4/5)	SOC	0.75			
SCQ 36—interested in other children (4/5)	SOC	0.75			
SCQ 37—positive response to other children (4/5)	SOC	0.72			
SCQ 34—joins in social games (4/5)	COM	0.71			
SCQ 21—spontaneously copies others actions (4/5)	COM	0.70			
SCQ 40—plays cooperatively with others (4/5)	SOC	0.67	0.32		
SCQ 26—looks at faces (4/5)	SOC	0.59			
SCQ 38—pays attention without name being called (4/5)	SOC	0.55	0.36		
SCQ 19—has friends	SOC	0.53			
SCQ 11—odd interests	RRB		0.79		
SCQ 13—intense interests	RRB		0.75		
SCQ 18—has to carry around specific object	RRB		0.74		
SCQ 12—interested in parts of objects	RRB		0.73		
SCQ 14—unusual sensory interests	RRB		0.70		
SCQ 6—makes up words	COM		0.70		
SCQ 3—uses odd phrases	RRB		0.68		
SCQ 15—odd movements	RRB		0.65		
SCQ 10—uses other’s hand as tool	SOC		0.64		
SCQ 16—repetitive complicated movements	RRB		0.64		
SCQ 8—has rituals	RRB		0.63		
SCQ 5—mixes up pronouns	COM		0.62		
SCQ 4—asks socially inappropriate questions	SOC		0.60		
SCQ 17—engages in self-harm	RRB		0.44		
SWAN 14—control constant activity	HYP/IMP				0.82
SWAN 13—plays quietly	HYP/IMP				0.79
SWAN 17—awaits turn	HYP/IMP				0.79
SWAN 15—controls excess talking	HYP/IMP				0.77
SWAN 16—controls blurting out answering	HYP/IMP				0.77
SWAN 10—sits still	HYP/IMP				0.67
SWAN 12—modulates motor activity	HYP/IMP				0.65
SWAN 5—organizes tasks	INA			0.85	
SWAN 7—loses things	INA			0.81	
SWAN 4—follows through on instructions	INA			0.73	
SWAN 9—forgetfulness	INA			0.69	
SWAN 6—engages in tasks requiring mental effort	INA			0.69	
SWAN 1—attention to detail	INA			0.69	
SWAN 2—sustains attention	INA			0.61	
SWAN 3—listens when spoken to	INA			0.46	0.35
SWAN 8—easily distracted	INA			0.38	

Component loadings above 0.3 are shown

*ADHD* attention-deficit/hyperactivity disorder, *ASD* autism spectrum disorder, *SCQ* social communication questionnaire, *SWAN* strengths and weaknesses of ADHD symptoms and normal behavior questionnaire, *RRBI* restricted repetitive behaviors and interests, *INA* inattention, *HYP/IMP* hyperactivity/impulsivity

### Component correlations

The “social-communication” component had a moderate correlation with the “RRBI” component (0.41), and low correlations with the “inattentive” and “hyperactivity/impulsivity” components, (0.11 and 0.10, respectively). The “RRBI” component had a low correlation with the “hyperactivity-impulsivity” component (0.17) and minimal correlation with the “inattentive” component (< 0.01). The “hyperactivity-impulsivity” component showed a moderate correlation with the “inattentive” component (0.34).

### Component scores

**Fig. 1:**
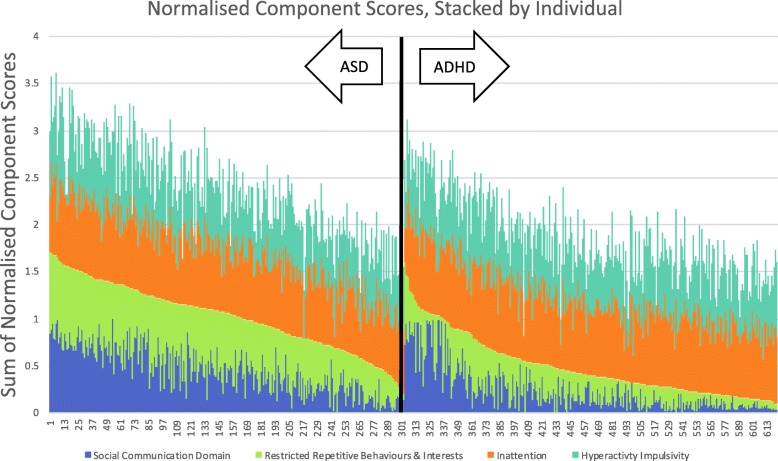
Normalized component scores, stacked by individual. Normalized component scores are summed for each individual in this stacked graph. Each child can have a score between 0 and 1 for each of the four components. Data are sorted from largest to smallest based on the sum of the social-communication and RRBI symptom domain component scores within each diagnosis

The sum of the normalized component scores for each domain for each participant is shown in Fig. 1. Each child could have a score on the component domain that ranged from 0 to 1.0 or a total score ranging from 0 to 4.0. Figure 1 shows that the component scores generally line up with the clinical diagnosis. However, there seems to be a sub-group of ADHD children with high social-communication component scores. A total of 11.91% of ADHD children had normalized social-communication component scores > 0.5 in this domain. In comparison, 42.24% of ASD children had a normalized component score of > 0.5 in this domain.

### Profile analysis

Overall, mean levels were not equal by diagnosis (F (1620) = 174.26, *p* < 0.001) or by gender (F (1620) = 613.63, *p* < 0.001), and there was no interaction between diagnosis and gender (F (1618) = 0.91, *p* = 0.34) with regard to overall levels of all four combined components. The tests of levels indicate that across all four components, there was a higher overall level of total scores in children in ASD than ADHD and in males than females. The test of parallel lines was significant for symptom components by diagnosis (F (3618) = 139.93, *p* < 0.001) and for symptom components by gender (F (3616) = 2.64, *p* = 0.05), but not for the interaction between symptom components by diagnosis by gender (F (3616) = 0.07, *p* = 0.97). The test of parallel lines indicates that the groups are not parallel by diagnosis, meaning that ADHD is not simply a milder version of ASD with lower levels of all symptom domains. Similarly, genders are not parallel, indicating that females are not scoring equally lower in all domains compared to males. The test of flatness was significant for diagnosis (F (31854) = 88.72, *p* < 0.001), but not for gender (F (31854) = 2.06, *p* = 0.10) and there was no interaction between diagnosis and gender (F (31854) = 0.06, *p* = 0.98). The test of flatness indicates that the diagnostic groups have significantly different amounts of one or more symptom components than that of other symptom components. The non-significant effect of gender and the interaction term indicates that it is the diagnosis, more so than gender, that drives the differences in the individual levels of the four symptom components.

**Fig. 2:**
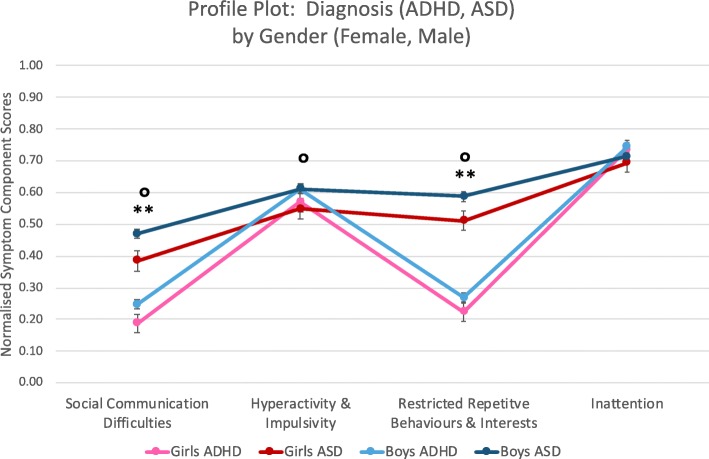
Profile plot with diagnosis by gender. There was no difference in inattentive and hyperactive/impulsive component scores between the two diagnostic groups (*p* < 0.01). Males had higher social-communication, hyperactivity/impulsivity, and RRBI component scores regardless of diagnosis (*p* < 0.01). Two asterisks (**) indicate a diagnosis effect. A degree sign (°) indicates a gender effect

A profile plot comparing component scores between diagnostic groups and genders across the components is shown in Fig. 2. Subsequent ANOVAs revealed that, while there was a significant difference in social-communication (F (1618) = 75.48, *p* < 0.01) and RRBI (F (1618) = 258.96, *p* < 0.01) component scores between the two diagnostic groups, there was no significant difference in inattentive (F (1618) = 5.67, *p* = 0.02) or hyperactive/impulsive (F (1618) = 0.31, *p* = 0.58) component scores between diagnostic groups. Males had higher social-communication (F (1618) = 9.10, *p* < 0.01), hyperactivity/impulsivity (F (1618) = 9.00, *p* < 0.01), and RRBI (F (1618) = 10.60, *p* < 0.01) component scores than females within each diagnostic group. There was no significant difference between males and females in inattentive (F (1618) = 0.93, *p* = 0.34) component scores.

## Discussion

High rates of co-occurrence between ASD and ADHD have been reported [5, 6]. To date, few studies have examined the underlying relationship between the core domains of combined ASD and ADHD symptoms. Our PCA revealed a four-component solution with no overlap between SCQ and SWAN items in the components. Children with ASD had higher scores in social-communication and RRBI components than children with ADHD, while there was no difference in inattentive and hyperactive/impulsive component scores between diagnostic groups. Males had higher component scores than females in social-communication, RRBI, and hyperactivity/impulsivity within each diagnostic category.

In our sample, while 53.14% of children with a primary diagnosis of ASD met the threshold for ADHD caseness on the SWAN, only 18.97% were listed as having a known co-occurring ADHD diagnosis. While 13.48% of children with a primary diagnosis of ADHD met the threshold for ASD caseness on the SCQ, only 1.36% were listed as having a known co-occurring ASD diagnosis. This suggests that co-occurring ASD in children with ADHD (and vice versa) may be under-identified and highlights the importance of assessing for co-occurring symptomatology in this population. Since the SCQ and SWAN were filled out by caregivers it is also important to consider that rates of ASD and ADHD caseness on the SCQ and SWAN, respectively, may have been overrepresented in our sample.

Even though ASD has an earlier age of onset than ADHD, studies have shown that many children with ASD are first diagnosed with having ADHD, subsequently leading to a delay in a diagnosis of ASD [41, 42]. This can have important consequences for access to services. Clinically, ADHD symptoms can overshadow those of ASD or clinicians may “expect” to see a more common disorder such as ADHD and might not carry out a careful assessment of other diagnoses. While National Institute for Health Care and Excellence ADHD guidelines caution that there are high levels of ADHD in children with ASD [43], the American Academy of Child and Adolescent Psychiatry ADHD practice parameter guidelines do not mention ASD [44] in the differential diagnosis. Based on our findings, co-occurring ASD in children with ADHD may be under-identified and we suggest that the importance of assessing for co-occurring ASD symptomatology in the ADHD population be considered in future guideline revisions.

When conducting a PCA on combined ASD and ADHD symptom measures in a sample of children with a primary diagnosis of ASD or ADHD, we found no overlap between ASD and ADHD items comprising the components. SCQ items loaded onto two ASD components (social-communication and RRBI) and SWAN items loaded onto two ADHD components (inattention and hyperactivity/impulsivity). Therefore, the explanation for the co-occurrence between the two disorders cannot simply be due to an overlap in their core domains. Also, there were low correlations between domains, so it is not a matter of scoring high on all components, which might reflect reporting bias. This suggests that the measurement structure cannot explain the covariation between the two disorders in clinical samples. We cannot assess “true comorbidity” based on the present findings since parent report bias has not been addressed in this study.

Our findings are in contrast to the study by Martin et al. [19] which found overlap between hyperactivity/impulsivity and RRBI domains in a sample of children with ADHD. Although we used PCA instead of exploratory factor analysis, both our study and the Martin et al. [19] study used an oblique rotation (allowing components to correlate) and the SCQ as a measure of ASD symptoms. Interestingly, in their sample of ADHD children, Martin et al. [19] reported finding another factor solution in which ASD and ADHD did not overlap, and consisted of three ASD factors and two ADHD factors. Such a finding is in line with the results of our study, which also found that ASD and ADHD items loaded independently onto separate components. Of note, Martin et al. [19] chose the competing three-factor solution composed of a social (ASD) factor, an inattentive (ADHD) factor, and a factor in which RRBI (ASD) and hyperactivity/impulsivity (ADHD) traits grouped together because it had a lower range of factor inter-correlations and greater parsimony. It is also important to consider that our sample had a relatively higher IQ score than Martin et al.’s. Given that IQ is negatively correlated with ASD and ADHD symptoms [45, 46], it is unclear how much IQ may have explained the difference in findings between the two studies.

Interestingly, our PCA of ASD children (Supplemental Table 3) revealed cross-loading of a few ASD items onto the ADHD hyperactivity/impulsivity component, and our PCA of ADHD children (Supplemental Table 4) revealed cross-loading of one ASD item onto the ADHD hyperactivity/impulsivity component. Since these items suggest potential misattribution of ASD traits for those of ADHD, and vice versa, we re-ran the PCA without these items, as a sensitivity analysis, to determine whether it resulted in a model with more variance explained. This PCA produced a similar model, with four components with similar variance explained (58 % vs. 55% in the original model).

In our study, 11.91% of ADHD children had normalized component scores > 0.5 in the social-communication domain. This agrees with latent class analysis studies that have shown considerable heterogeneity when looking at the intersection of ASD and ADHD symptoms across subgroups [47–49]. Given the heterogeneity seen in ADHD, it is possible that there is a subgroup of ADHD children with significant and impairing problems in social-communication without accompanying RRBI, similar to the DSM-5 social (pragmatic) communication disorder or the so-called broader autism phenotype [50, 51] seen in first degree relatives of probands with ASD. Further study of this group might prove revealing in terms of biomarker and genetic variant overlap with ASD.

Using profile analysis, our study showed that there was no difference in inattentive and hyperactivity component scores between ASD and ADHD groups. This suggests a possible mechanism for the increasing literature showing an overlap between ASD and ADHD on genetic and neuroimaging levels [7, 52]. This overlap might be accounted for by similar levels of inattentive and hyperactive/impulsive phenotypes in the two groups instead of variation in social-communication and RRBI.

By finding high levels of inattention and hyperactivity/impulsivity in ASD and ADHD, our study supports the need for a dimensional framework that examines neurodevelopmental domains (social-communication, RRBI, inattention, and hyperactivity/impulsivity) across traditional ASD and ADHD diagnostic boundaries. This notion is also supported by recent neuroimaging studies in children with ASD and ADHD [53–55]. In the POND cohort, Baribeau et al. [55] found that structural neuroimaging correlates of social deficits were similar across ASD and ADHD, and Kushki et al. [54] found that clusters identified among children with ASD, ADHD, and OCD based on cortical thickness and behavioral phenotypic features did not map well onto traditional diagnostic categories. In a separate cohort, Aoki et al. [53] further showed that ASD severity across both children with ASD or ADHD was associated with specific white matter organization indices and that a dimensional approach provided a more comprehensive picture of white matter associations than a categorical approach. Our study adds to the literature by showing that ASD and ADHD groups differ on component scores in social-communication and RRBI domains, suggesting that some domains may be relatively more disorder-specific and may be useful in identifying certain subgroups in ASD and ADHD.

We also found that females had lower component scores across social-communication, RRBI, and hyperactivity/impulsivity domains. This suggests that there may be gender-related phenotypes associated with the two neurodevelopmental disorders. A “female phenotype” of ASD has been proposed in the literature [22], one aspect being that females generally show less RRBIs than males [56]. However, it has also been argued that “gold standard” diagnostic instruments are potentially male-biased and RRBIs that are more prevalent in females may be harder to capture using these instruments. Females may also show different forms of social-communication difficulties than males, being better able to express themselves socially but having similar levels of social difficulties as males [56, 57]. This suggests that females may tend to have a better ability to “camouflage” or compensate their autism, for example, by imitating social scripts [58, 59]. In terms of ADHD, females tend to have less hyperactive symptoms and externalizing behaviors than males [23], which agrees with our findings that females have lower component scores in the hyperactivity/impulsivity domain than males.

Interestingly, when looking at factor scores across genders in their combined ASD-ADHD symptom analysis, Martin et al. [19] did not find any differences in “social,” “rigidity-hyperactivity,” or “inattentiveness” domains between males and females. Compared to this study, our participants had higher IQ; therefore, it is possible that females in this group were better able to “camouflage” or compensate their ASD characteristics, hence such presentations were less noticeable and therefore scored lower by caregivers. ADHD characteristics in females with higher IQ may also be less noticeable due to compensation of the child or recognition biases of caregivers, especially if there are fewer concerns about their school performance. These influences may have widened the difference in social-communication, RRBI, and hyperactivity/impulsivity component scores between males and females in our sample.

The strengths of our study include the large sample size of participants with ASD (*n* = 303) and ADHD (*n* = 319). The primary clinical diagnoses of all the study participants were also confirmed with valid and reliable measures: the PICS for ADHD [27] and the ADOS-2 [24] and ADI-R [26] for ASD. Our study also used a combined sample of children with a primary diagnosis of ASD or ADHD which provides a larger sample size and a more robust test of the underlying relationship between the core domains of ASD and ADHD symptoms than a sample of children with ASD or ADHD alone.

## Limitations

Our study had several limitations. First, we did not perform the ASD diagnostic interviews on the clinically referred ADHD participants, and we did not perform an ADHD diagnostic interview on the clinically referred ASD participants. It is therefore unclear which ASD and ADHD participants had a co-occurring ADHD or ASD diagnosis, respectively, defined using the same research criteria. Such information would be helpful as an increased number of ASD participants with co-occurring ADHD might increase levels of inattention and hyperactivity/impulsivity and an increased number of ADHD participants with co-occurring ASD might increase levels of social-communication and RRBI symptoms. Future studies should consider comparing ASD, ADHD, and co-occurring ASD + ADHD groups separately when examining neurodevelopmental domain scores. Although our study did collect information from caregivers about co-occurring diagnoses, including ASD and ADHD, it is unclear if this information is sufficiently valid, and many participants may not have been assessed for co-occurring diagnoses or may have been misdiagnosed. It is important for future studies to formally assess for co-occurring diagnoses using validated instruments across all participants.

Second, we used parent-rated questionnaires. A disadvantage with using single informant-based reports is that some ASD and ADHD symptoms may be difficult to distinguish by parents, and may be interpreted differently between parents and clinicians [60].

Third, we excluded participants with missing data on the rating scales (which included non-verbal or minimally verbal participants). Children with incomplete rating scales had significantly lower IQ and ABAS scores, which likely resulted in a biased sample of participants for our PCA.

Finally, we combined two different rating scales with continuous (SWAN) and dichotomous (SCQ) variables in the PCA. It is therefore unclear to what extent our findings might be influenced by items from the same questionnaire grouping together as a result of being in the same measurement scale. It would be interesting to conduct a PCA on ASD and ADHD items from the same questionnaire, such as the child behavior checklist (CBCL); however, such questionnaires are typically based on broad DSM-based criteria and as such do not adequately capture ASD symptomatology.

## Conclusions

ASD and ADHD items loaded on separate components in our sample, suggesting that the measurement structure cannot explain the covariation between the two disorders in clinical samples. Our results also show that high levels of inattention and hyperactivity/impulsivity are seen in both ASD and ADHD. This supports the need for a dimensional framework that examines neurodevelopmental domains across traditional diagnostic boundaries. Clinically, this emphasizes the importance of careful screening for social communication difficulties and RRBIs in children presenting with high levels of inattention and hyperactivity/impulsivity. Moreover, females had lower component scores across social-communication, RRBI, and hyperactivity/impulsivity than males, suggesting that there may be gender-related phenotypes in the two neurodevelopmental conditions. An increasing amount of literature suggests that females show fewer observable difficulties than males, secondary to their tendency to “camouflage” or compensate for difficulties. This highlights the need for careful screening of ASD symptomatology in females, especially those with ADHD.

## Supplementary information

**Additional file 1: Table S1.** Comparison of complete versus missing data for ASD study sample. **Table S2.** Comparison of complete versus missing data for ADHD study sample. **Table S3.** Pattern matrix of PCA loadings of SCQ and SWAN items in ASD sample. **Table S4.** Pattern matrix of PCA loadings of SCQ and SWAN items in ADHD sample.

## Data Availability

The datasets generated and analyzed during the current study are publicly available through Brain-CODE. https://www.braincode.ca/
