# Supplementary material for: Inattention and hyperactive/impulsive component scores do not differentiate between autism spectrum disorder and attention-deficit/hyperactivity disorder in a clinical sample
Source: Mol Autism. 2020 Apr 25;11:28. doi: 10.1186/s13229-020-00338-1 (PMC7183643; doi:10.1186/s13229-020-00338-1)
Supplement: Supplementary file 1 — Additional file 1: Table S1. Comparison of complete versus missing data for ASD study sample. Table S2. Comparison of complete versus missing data for ADHD study sample. Table S3. Pattern matrix of PCA loadings of SCQ and SWAN items in ASD sample. Table S4. Pattern matrix of PCA loadings of SCQ and SWAN items in ADHD sample. [file 13229_2020_338_MOESM1_ESM.docx]

Supplemental Table 1: Comparison of complete versus missing data for ASD study sample

|  | Complete  (n=303) | Missing  (n=136) |  |  |
| --- | --- | --- | --- | --- |
|  | Mean (SD) | Mean (SD) | *p-value* | *Cohen’s d* |
| Age (years) | 11.22 (3.43) | 11.18 (3.78) | 0.91 | 0.01 |
| IQ^a^ | 87.49 (24.63) | 79.64 (30.95) | 0.02 * | 0.28 |
| ABAS Composite Scores^b^:    Conceptual:  Social:  Practical: | 71.07 (15.36)  70.96 (12.43)  64.23 (18.35) | 63.14 (16.06)  66.02 (13.67)  58.51 (20.54) | <0.001**  <0.001**  <0.01 ** | 0.51  0.38  0.29 |
| SCQ score | 19.96 (7.86) | 21.03 (6.63) | 0.14 | 0.15 |
| SWAN INA score | 4.72 (2.94) | 5.13 (3.18) | 0.20 | 0.13 |
| SWAN IMP/HYP score | 3.68 (2.96) | 4.29 (3.19) | 0.05 | 0.20 |
|  |  |  |  |  |
|  | *n* (%) | *n* (%) | *p-value* |  |
| Males | 242 (79.87 %) | 105 (77.21 %) | 0.53 |  |

*ADHD* attention-deficit/hyperactivity disorder, *ASD* autism spectrum disorder, *ABAS* adaptive behavior assessment system, *SCQ* social communication questionnaire, *SWAN* strengths and weaknesses of ADHD symptoms and normal behaviour questionnaire, ^a^ 30 participants with complete questionnaire data were missing IQ information and 37 participants with missing questionnaire data were missing IQ information, ^b^ 21 participants with complete questionnaire data were missing ABAS information and 11 participants with missing questionnaire data were missing ABAS information * p <0.05 ** p <0.01

Supplemental Table 2: Comparison of complete versus missing data for ADHD study sample

|  | Complete  (n=319) | Missing  (n=106) |  |  |
| --- | --- | --- | --- | --- |
|  | Mean (SD) | Mean (SD) | *p-value* | *Cohen’s d* |
| Age (years) | 10.08 (2.74) | 9.39 (2.91) | 0.03 * | 0.24 |
| IQ^a^ | 97.50 (15.91) | 101.64 (17.32) | 0.14 | 0.25 |
| ABAS Composite Scores^b^:    Conceptual:  Social:  Practical: | 81.38 (14.04)  85.68 (16.52)  79.49(17.19) | 82.91 (16.54)  86.27 (16.87)  79.73 (17.96) | 0.48  0.81  0.92 | 0.10  0.04  0.01 |
| SCQ score | 7.76 (5.94) | 8.35 (6.55) | 0.39 | 0.09 |
| SWAN INA score | 5.40 (2.88) | 5.08 (3.01) | 0.33 | 0.11 |
| SWAN IMP/HYP score | 3.49 (3.10) | 3.94 (2.98) | 0.19 | 0.15 |
|  |  |  |  |  |
|  | *n* (%) | *n* (%) | *p-value* |  |
| Males | 253 (79.31 %) | 81 (76.42 %) | 0.59 |  |

*ADHD* attention-deficit/hyperactivity disorder, *ASD* autism spectrum disorder, *ABAS* adaptive behavior assessment system, *SCQ* social communication questionnaire, *SWAN* strengths and weaknesses of ADHD symptoms and normal behaviour questionnaire, ^a^ 193 participants with complete questionnaire data were missing IQ information and 59 participants with missing questionnaire data were missing IQ information, ^b^ 131 participants with complete questionnaire data were missing ABAS information and 42 participants with missing questionnaire data were missing ABAS information * p <0.05 ** p <0.01

Supplemental Table 3: Pattern matrix of PCA loadings of SCQ and SWAN items in ASD sample

| Items | DSM-IV  sub-domains | Component 1- SOC/COM | Component 2-  HYP/IMP | Component 3-  RRBI | Component 4-  INA |
| --- | --- | --- | --- | --- | --- |
| SCQ 20- talk to be friendly (4/5) | COM | 0.80 |  |  |  |
| SCQ 28- show things to engage interest (4/5) | SOC | 0.75 |  |  |  |
| SCQ 36- interested in other children (4/5) | SOC | 0.75 |  |  |  |
| SCQ 27- reciprocates smiles (4/5) | SOC | 0.74 |  |  |  |
| SCQ 29- shares things (4/5) | SOC | 0.73 |  |  |  |
| SCQ 35- pretend play (4/5) | COM | 0.73 |  |  |  |
| SCQ 33- normal range of facial expressions (4/5) | SOC | 0.69 |  |  |  |
| SCQ 34- joins in social games (4/5) | COM | 0.68 |  |  |  |
| SCQ 40- plays cooperatively with others (4/5) | SOC | 0.67 |  |  |  |
| SCQ 31- comforts others (4/5) | SOC | 0.66 |  |  |  |
| SCQ 22- points to show things (4/5) | COM | 0.66 |  |  |  |
| SCQ 37- positive response to other children (4/5) | SOC | 0.64 |  |  |  |
| SCQ 24- nod head (4/5) | COM | 0.62 |  |  |  |
| SCQ 21- spontaneously copies others actions (4/5) | COM | 0.60 |  |  |  |
| SCQ 26- looks at faces (4/5) | SOC | 0.58 |  |  |  |
| SCQ 32- use gestures with sounds or words (4/5) | SOC | 0.58 |  |  |  |
| SCQ 38- pays attention without name being called (4/5) | SOC | 0.53 |  |  |  |
| SCQ 19- has friends | SOC | 0.34 |  |  |  |
| SWAN 14- control constant activity | HYP/IMP |  | 0.79 |  |  |
| SWAN 13- plays quietly | HYP/IMP |  | 0.76 |  |  |
| SWAN 17- awaits turn | HYP/IMP |  | 0.74 |  |  |
| SWAN 16- controls blurting out answering | HYP/IMP |  | 0.73 |  |  |
| SWAN 10- sits still | HYP/IMP |  | 0.68 |  |  |
| SWAN 15- controls excess talking | HYP/IMP |  | 0.67 |  |  |
| SWAN 12- modulates motor activity | HYP/IMP |  | 0.63 |  |  |
| SCQ 16- repetitive complicated movements | RRB |  | 0.53 | 0.38 |  |
| SCQ 2- has to and fro conversation | COM | 0.44 | 0.49 |  |  |
| SCQ 17- engages in self-harm | RRB |  | 0.41 |  |  |
| SWAN 8- easily distracted | INA |  | 0.37 |  |  |
| SCQ 6- makes up words | COM |  |  | 0.67 | 0.36 |
| SCQ 18- has to carry around specific object | RRB |  |  | 0.66 |  |
| SCQ 11- odd interests | RRB |  |  | 0.63 |  |
| SCQ 13- intense interests | RRB |  |  | 0.62 |  |
| SCQ 3- uses odd phrases | RRB |  |  | 0.58 |  |
| SCQ 14- unusual sensory interests | RRB |  |  | 0.57 |  |
| SCQ 12- interested in parts of objects | RRB |  |  | 0.55 |  |
| SCQ 8- has rituals | RRB |  |  | 0.53 |  |
| SCQ 4- asks socially inappropriate questions | SOC |  |  | 0.49 |  |
| SCQ 5- mixes up pronouns | COM |  |  | 0.46 |  |
| SCQ 10- uses other’s hand as tool | SOC |  |  | 0.44 |  |
| SCQ 15- odd movements | RRB |  |  | 0.37 |  |
| SWAN 5- organizes tasks | INA |  |  |  | 0.77 |
| SWAN 7- loses things | INA |  |  |  | 0.72 |
| SWAN 6- engages in tasks requiring mental effort | INA |  |  |  | 0.72 |
| SWAN 4-follows through on instructions | INA |  |  |  | 0.66 |
| SWAN 2- sustains attention | INA |  |  |  | 0.65 |
| SWAN 1- attention to detail | INA |  |  |  | 0.62 |
| SWAN 9- forgetfulness | INA |  |  |  | 0.53 |
| SWAN 3- listens when spoken to | INA |  |  |  | 0.47 |
| SWAN 18- enter into conversations and games | HYP/IMP |  |  |  | 0.38 |

Component loadings above 0.3 are shown.

*ADHD* attention-deficit/hyperactivity disorder, *ASD* autism spectrum disorder, *SCQ* social communication questionnaire, *SWAN* strengths and weaknesses of ADHD symptoms and normal behaviour questionnaire, *RRBI* restricted repetitive behaviours and interests, *INA* inattention, *HYP/IMP* hyperactivity/impulsivity

Supplemental Table 4: Pattern matrix of PCA loadings of SCQ and SWAN items in ADHD sample

| Items | DSM-IV  sub-domains | Component 1- SOC/COM | Component 2-  HYP/IMP | Component 3-  RRBI | Component 4-  INA |
| --- | --- | --- | --- | --- | --- |
| SCQ 28- show things to engage interest (4/5) | SOC | 0.96 |  |  |  |
| SCQ 2- has to and fro conversation | COM | 0.89 |  |  |  |
| SCQ 33- normal range of facial expressions (4/5) | SOC | 0.88 |  |  |  |
| SCQ 27- reciprocates smiles (4/5) | SOC | 0.87 |  |  |  |
| SCQ 31- comforts others (4/5) | SOC | 0.87 |  |  |  |
| SCQ 29- shares things (4/5) | SOC | 0.86 |  |  |  |
| SCQ 37- positive response to other children (4/5) | SOC | 0.82 |  |  |  |
| SCQ 32- use gestures with sounds or words (4/5) | SOC | 0.82 |  |  |  |
| SCQ 24- nod head (4/5) | COM | 0.82 |  |  |  |
| SCQ 20- talk to be friendly (4/5) | COM | 0.81 |  |  |  |
| SCQ 36- interested in other children (4/5) | SOC | 0.79 |  |  |  |
| SCQ 35- pretend play (4/5) | COM | 0.77 |  |  |  |
| SCQ 34- joins in social games (4/5) | COM | 0.76 |  |  |  |
| SCQ 22- points to show things (4/5) | COM | 0.74 |  |  |  |
| SCQ 40- plays cooperatively with others (4/5) | SOC | 0.73 |  |  |  |
| SCQ 21- spontaneously copies others actions (4/5) | COM | 0.69 |  |  |  |
| SCQ 38- pays attention without name being called (4/5) | SOC | 0.62 |  |  |  |
| SCQ 26- looks at faces (4/5) | SOC | 0.59 |  |  |  |
| SCQ 19- has friends | SOC | 0.55 |  |  |  |
| SCQ 11- odd interests | RRB |  | 0.78 |  |  |
| SCQ 12- interested in parts of objects | RRB |  | 0.71 |  |  |
| SCQ 14- unusual sensory interests | RRB |  | 0.68 |  |  |
| SCQ 13- intense interests | RRB |  | 0.67 |  |  |
| SCQ 10- uses other’s hand as tool | SOC |  | 0.64 |  |  |
| SCQ 16- repetitive complicated movements | RRB |  | 0.61 |  |  |
| SCQ 18- has to carry around specific object | RRB |  | 0.59 |  |  |
| SCQ 6- makes up words | COM |  | 0.58 |  |  |
| SCQ 15- odd movements | RRB |  | 0.58 |  |  |
| SCQ 8- has rituals | RRB |  | 0.55 |  |  |
| SCQ 5- mixes up pronouns | COM |  | 0.52 |  |  |
| SCQ 3- uses odd phrases | RRB |  | 0.51 |  |  |
| SCQ 17- engages in self-harm | RRB |  | 0.37 |  |  |
| SWAN 17- awaits turn | HYP/IMP |  |  | 0.84 |  |
| SWAN 14- control constant activity | HYP/IMP |  |  | 0.83 |  |
| SWAN 15- controls excess talking | HYP/IMP |  |  | 0.78 |  |
| SWAN 16- controls blurting out answering | HYP/IMP |  |  | 0.76 |  |
| SWAN 13- plays quietly | HYP/IMP |  |  | 0.76 |  |
| SWAN 10- sits still | HYP/IMP |  |  | 0.63 |  |
| SWAN 12- modulates motor activity | HYP/IMP |  |  | 0.63 |  |
| SWAN 18- enter into conversations and games | HYP/IMP |  |  | 0.43 |  |
| SCQ 4- asks socially inappropriate questions | SOC |  | 0.36 | 0.40 |  |
| SWAN 5- organizes tasks | INA |  |  |  | 0.85 |
| SWAN 7- loses things | INA |  |  |  | 0.81 |
| SWAN 9- forgetfulness | INA |  |  |  | 0.77 |
| SWAN 4-follows through on instructions | INA |  |  |  | 0.76 |
| SWAN 1- attention to detail | INA |  |  |  | 0.70 |
| SWAN 6- engages in tasks requiring mental effort | INA |  |  |  | 0.63 |
| SWAN 2- sustains attention | INA |  |  |  | 0.62 |
| SWAN 8- easily distracted | INA |  |  |  | 0.46 |
| SWAN 3- listens when spoken to | INA |  |  |  | 0.45 |

Component loadings above 0.3 are shown.

*ADHD* attention-deficit/hyperactivity disorder, *ASD* autism spectrum disorder, *SCQ* social communication questionnaire, *SWAN* strengths and weaknesses of ADHD symptoms and normal behaviour questionnaire, *RRBI* restricted repetitive behaviours and interests, *INA* inattention, *HYP/IMP* hyperactivity/impulsivity
